# Establishment and Implementation of the Point-of-Care RT-RAA-CRISPR/Cas13a Diagnostic Test for Foot-And-Mouth Disease Virus Serotype O in Pigs

**DOI:** 10.3390/v17050721

**Published:** 2025-05-17

**Authors:** Ping Meng, Bo Ni, Chenyu Li, Zhou Sha, Chunju Liu, Weijie Ren, Rong Wei, Fuxiao Liu, Jinming Li, Zhiliang Wang

**Affiliations:** 1 China Animal Health and Epidemiology Center, Qingdao 266011, China; 19170578305@163.com (P.M.); nibo@cahec.cn (B.N.); shazhou@cahec.cn (Z.S.); liuchunju@cahec.cn (C.L.); renweijie@cahec.cn (W.R.); weirong@cahec.cn (R.W.); 2 College of Veterinary Medicine, Qingdao Agricultural University, Qingdao 266109, China; laudawn@126.com; 3 College of Veterinary Medicine, Shanxi Agricultural University, Jinzhong 030031, China; 15392552348@163.com

**Keywords:** point-of-care diagnostic test, RT-RAA, CRISPR/Cas13a, FMDV serotype O

## Abstract

Foot and mouth disease virus (FMDV) is a highly pathogenic virus that mainly infects cloven hooved animals, such as pigs. The establishment of a rapid, sensitive and accurate point-of-care detection method is critical for the timely identification and elimination of infected pigs for controlling this disease. In this study, a RT-RAA-CRISPR/Cas13a method was developed for the detection of FMDV serotype O in pigs. Six pairs of RT-RAA primers were designed based on the conserved gene sequence of FMDV serotype O, and the optimal amplification primers and reaction temperatures were screened. The CRISPR-derived RNA (crRNA) was further designed based on the optimal target band sequence and the most efficient crRNA was screened. The results revealed that FMDV-O-F4/R4 was the optimal primer set, and the optimal temperature for the RT-RAA reaction was 37 °C. Moreover, crRNA4 exhibited the strongest detection signal among the six crRNAs. The established RT-RAA-CRISPR/Cas13a method demonstrated high specificity and no cross-reactivity with other common swine pathogens such as Senecavirus A (SVA), porcine reproductive and respiratory virus (PRRSV), porcine epidemic diarrhea virus (PEDV), porcine circovirus type 2 (PCV2), classical swine fever virus (CSFV), and pseudorabies virus (PRV), additionally, it was observed to be highly sensitive, with a detection limit of 19.1 copies/µL. The repeatability of this method was also observed to be good. This method could produce stable fluorescence and exhibited good repeatability when three independent experiments yielded the same results. A validation test using three types of simulated clinical samples (including swab, tissue, and serum samples) revealed a 100% concordance rate. The detection results could be visualized via a fluorescence reader or lateral flow strips (LFSs). Thus, a highly specific and sensitive RT-RAA-CRISPR/Cas13a detection method was developed and is expected to be applied for the rapid detection of FMDV serotype O in situ.

## 1. Introduction

Foot-and-mouth disease (FMD) is a severe infectious disease caused by the foot-and-mouth disease virus (FMDV), which is considered to be one of the most severe diseases endangering cloven-hoofed animals, such as pigs, cattle, and sheep [1]. The disease is extremely contagious and spreads rapidly [2]. FMD is considered as one of the most contagious animal diseases by the World Organization for Animal Health (WOAH) [3]. FMDV belongs to the *Picornaviridae* [4]. Additionally, it possesses a single-stranded positive-strand RNA genome with a total length of approximately 8500 nucleotides (nt) [5]. The viral particle is a nonenveloped icosahedron with a diameter of approximately 25–30 nm [6]. There are currently seven serotypes of FMDV, including Type A, Type O, Type C, Asian Type I (AsiaI), South African Type I (SAT1), South African Type II (SAT2), and South African Type III (SAT3) [2]. In China, Type O and Type A FMDVs are currently present, of which Type O FMDV is the most prevalent [7]; moreover, the strains of serotype O FMDVs in pigs are mainly divided into two subtypes: Cathay and SEA [8]. FMDV can cause illness in pig herds of different species and ages. Additionally, it can be transmitted both by infected animals and in the form of aerosols that spread over long distances [9]. The clinical symptoms of FMD are characterized by blisters and ulcerations on the pig’s oral mucosa, nose, hooves, and breast skin [10,11], which are very similar to the vesicular symptoms caused by other pathogens, such as swine vesicular disease virus (SVDV), vesicular stomatitis virus (VSV), vesicular exanthema of swine virus (VESV), and Seneca virus A (SVA). Panic has been observed to quickly spread among breeding groups after the occurrence of disease outbreaks [12]. At present, the differential diagnosis of porcine vesicular diseases can only be achieved via laboratory testing. Therefore, convenient, sensitive, accurate and rapid detection technology for FMDV serotype O is crucial for preventing and controlling its spread.

With the development of diagnostic technologies, multiple detection technologies have been applied, including fluorescent quantitative RT-PCR [13], enzyme-linked immunosorbent assays [14], reverse transcription loop-mediated isothermal amplification [15], and so on [16]. These methods are time-consuming and labor-intensive; moreover, they require favorable laboratory conditions, expensive instrumentation, and experienced technicians, which leads to difficulties in applying these methods in point-of-care testing. Although rapid detection technologies, such as loop-mediated isothermal amplification and recombinase polymerase amplification (RPA), can be used for point-of-care testing without the need for complex equipment, they have several shortcomings, such as low specificity and high false-positive rates [15,17]. Therefore, the establishment of a rapid FMDV serotype O detection method that is fast, accurate, and efficient is critically important.

The clustered regulatory interspaced short palindromic repeats/CRISPR-associated protein (CRISPR/Cas) system is a defense mechanism that exists in most bacteria and in all archaea; moreover, it is widely applicable in gene editing activities [18]. When the crRNA binds to the target RNA, the Cas13a protein undergoes conformational changes and its RNA cleavage activity can be activated, thus allowing it to nonspecifically cleave single-stranded RNA probes to generate fluorescent signals [19,20,21]. This system has been widely used in the field of viral RNA detection [22], and the viral RNA concentration is the key determinant of signal intensity. Therefore, an increase in the target copy number enhances the sensitivity of the assay [23,24,25]. In 2018, Zhang Feng et al. jointly applied RAA technology and CRISPR technology, thus effectively combining the high amplification strength of RAA and the high specificity of CRISPR [25].

**Figure 1:**
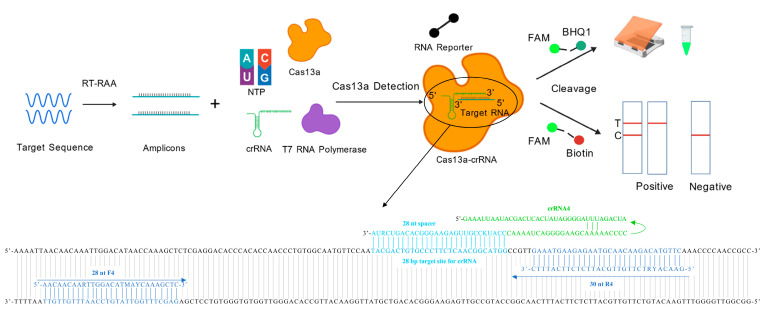
Schematic workflow of RT-RAA-CRISPR/Cas13a-based detection for porcine FMDV serotype O. The conserved gene of FMDV was amplified by RT-RAA, and the products were tested via the CRISPR/Cas13a detection system. After the Cas13a-crRNA complex is bound to the target RNA, the RNA reporter is cleaved. This cleavage generates a signal that can be directly superficially observed as green fluorescence under the blue light, alternatively, it can be measured using the LFS.

In this study, an FMDV serotype O RT-RAA-CRISPR/Cas13a detection method exhibiting high sensitivity, specificity, and repeatability was established to provide a new onsite diagnostic technology for FMDV control. Moreover, this technique can be easily applied to lateral flow strips (LFSs), which are very suitable for point-of-care testing (Figure 1).

## 2. Materials and Methods

### 2.1. Design of RT-RAA Primers, CRISPR crRNAs, Digital Droplet PCR Primers, Probes, and RNA Reporters

The complete genome sequences of 10 FMDV serotype O strains from China (2010~2024) or neighboring countries were downloaded from the GenBank database (Table S1) and aligned by using BioEdit (Figure S1). Six pairs of RT-RAA specific primers were designed based on the conserved sequence, and the T7 promoter sequence was introduced at the 5′ end of the upstream primer (Table 1). The 5′ and 3′ ends of the fluorescent RNA and LFS RNA reporter probes were modified as 5′ FAM/3′ BHQ1 and 5′ FAM/3′ Biotin, respectively (Table 1). The primers, probes, and crRNA templates were synthesized by Sangon Bioengineering Co., Ltd. (Shanghai, China).

### 2.2. Nucleic Acids and Samples

The nucleic acids of SVA, PRV, PRRSV, PEDV, PCV2, and CSFV were extracted from virus cultures preserved by the China Animal Health and Epidemiology Center. The FMDV serotype O RNA template was extracted from the FMDV O inactivated vaccine for pigs (Tecon, Urumqi, China). Pig tissue, serum, and swab samples were preserved by the China Animal Health and Epidemiology Center.

### 2.3. Screening of RT-RAA Primers and the Optimal Temperature for RT-RAA

The following RT-RAA reaction system parameters were utilized: 25 µL of buffer, 2 µL each of the upstream and downstream primers (10 µM), 5 µL of RNA template, and 5 µL of magnesium acetate I, with ddH_2_O being added to a total volume of 50 µL. Subsequently, the mixture was incubated at 37 °C for 40 min. The target sequence was detected via 2% agarose gel electrophoresis, and the primer pair that yielded a single, bright band after amplification was deemed to be the best RT-RAA primer pair. The tested temperature for optimizing the RT-RAA reaction were 37, 38, 39, 40, and 41 °C. The optimal reaction temperature was selected based on the results of 2% gel electrophoresis.

### 2.4. Preparation of crRNAs

Both the T7 oligo and crRNA templates 1–6 were used to generate crRNA as previously described [26]. Briefly, mixtures of the T7 oligo and crRNA template were incubated at 95 °C for 5 min for denaturation, and then slowly cooled to 10 °C. The cooling process occurred for no less than 56 min to facilitate the annealing and formation of double-stranded DNA. This double-stranded DNA was used as a template for in vitro transcription. The crRNA was synthesized in vitro at 37 °C for 2 h by using the T7 High Yield RNA Transcription Kit (Vazyme, Nanjing, China). The transcription product was subsequently treated with DNase I at 37 °C for 30 min to degrade the dsDNA template. The crRNA was purified with RNA isolation beads (SyNTHGENE, Nanjing, China) and stored at −80 °C until use.

### 2.5. Screening of the crRNAs and the RT-RAA-CRISPR/Cas13a Reaction System

Six crRNA templates (Table 1) were designed using the NCBI Primer-BLAST (http://www.ncbi.nlm.nih.gov/tools/primer-blast/ (accessed on 1 May 2025)) based on the optimal target band sequence, and were used for the CRISPR/Cas13a reaction. After the reaction was completed, the fluorescence intensity was measured, and the findings were used to identify the optimal crRNA. The following CRISPR/Cas13a reaction system parameters were utilized: 10× Cas13a reaction buffer (1 µL); crRNA4 (15 ng); Gen Crispr^TM^ Cas13a nuclease (200 ng); and ddH_2_O (up to 10 µL). Subsequently, the reaction was performed at 37 °C for 10 min. After 10 min, the original reaction mixture was supplemented with 5 µL of 10× Cas13a reaction buffer, 4 µL of rNTP mixture, 2 µL of RNase inhibitor, 1 µL of T7 RNA polymerase, 5 µL of reporter (10 µM), 5 µL of RT-RAA amplification product, and the addition of ddH_2_O (up to 60 µL). The samples were incubated in a metal bath at a constant temperature of 37 °C for 30 min; subsequently, blue light irradiation was used to observe the fluorescence results, and a multifunction microplate reader (Flash SuPerMax3100 SHANPU, Shanghai, China) was used to measure the fluorescence intensity.

### 2.6. Lateral Flow Assay

For the lateral flow assay, the fluorescence RNA reporter in the CRISPR/Cas13a reaction system was replaced with the LFS RNA reporter. The reaction products were diluted 1:1 in ddH_2_O, after which the commercial lateral flow strips (TOLOBIO, Shanghai, China) were used for detection. The reaction products were incubated with the lateral flow strips at room temperature for 10 min. The results were recorded with a camera.

### 2.7. RT-RAA-CRISPR/Cas13a Detection Method Specificity Test

CSFV, PEDV, PCV2, PRV, PRRSV, and SVA nucleic acids were used as reaction templates. The ddH_2_O was used as a negative control, and FMDV serotype O RNA was used as a positive control, RT-RAA-CRISPR/Cas13a detection was performed. The fluorescence intensity values and LFS results were measured.

### 2.8. Calibration of the Copy Number of FMDV Serotype O RNA by Digital PCR

The FMDV serotype O RNA template was subjected to 10-fold serial dilutions (1 × 10^0^–1 × 10^−10^). A digital PCR instrument (Pilot Gene) was used to calibrate the copy number of the standard FMDV RNA nucleic acid using the diluted RNA templates. The reaction program utilized the following parameters: reverse transcription at 55 °C for 15 min, followed by predenaturation at 95 °C for 30 s, as well as 45 cycles of 95 °C for 10 s, and 60 °C of amplification for 30 s. The reaction system consisted of 4 µL of 5× One Step U^+^ Mix, 1 µL of One Step U^+^ Enzyme Mix, 0.4 µL of 50× Rox Reference Dye 1, and 0.5 µL each of upstream and downstream primers and fluorescent probes (10 µM); this mixture system was diluted with 2 µL of FMDV RNA, and up to 20 µL ddH_2_O.

### 2.9. Sensitivity of the RT-RAA-CRISPR/Cas13a Detection Method

The calibrated FMDV serotype O RNA dilutions of 1 × 10^0^–1 × 10^−10^ were used as templates to conduct the RT-RAA-CRISPR/Cas13a reaction, and both the fluorescence intensity and LFS were measured to calculate the sensitivity of the method.

### 2.10. RT-RAA-CRISPR/Cas13a Detection Method Repeatability Test

Using the 10^−1^ and 10^−3^ dilutions of the FMDV serotype O RNA as templates and ddH_2_O as the negative control, three independent RT-RAA-CRISPR/Cas13a reactions were performed, and both the fluorescence intensity and LFS were measured, and used to test the repeatability of the method.

### 2.11. Simulated Clinical Samples Testing

Three types of samples (including swab, tissue, and serum samples) collected from healthy pigs (FMDV qRT-PCR-negative results) were used to prepare simulated clinical samples. Briefly, samples were mixed with approximately 1000 copies of each FMDV serotype O RNA samples to produce FMDV-positive simulated clinical samples. The RNA was extracted from all of the samples, and a blind test was conducted to assess the accuracy of the RT-RAA-CRISPR/Cas13a method for clinical sample detection.

### 2.12. Data Analysis

All of the data in this study were analyzed for significant differences via one-way ANOVA and plotted with GraphPad Prism 8 software.

## 3. Results

### 3.1. Screening of RT-RAA Primers

**Figure 2:**
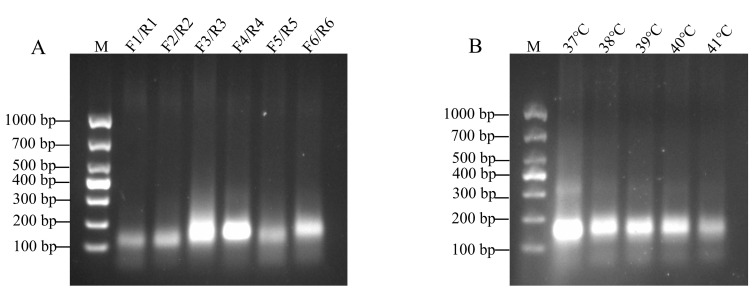
Gel electrophoresis results of the RT-RAA products. (**A**) Screening of the primer pairs. (**B**) Optimal RT-RAA reaction temperature screening. The temperature is marked at the top. M: DNA marker DL1000.

Six pairs of RT-RAA primers were designed based on the conserved sequences of FMDV serotype O, and the amplification efficiency was tested via the RT-RAA kit. The results of agarose gel electrophoresis (Figure 2A) revealed that the FMDV-O-F4/R4 primer set produced the brightest and most distinct amplification band, thus demonstrating the highest amplification efficiency. Therefore, the FMDV-O-F4/R4 primer set was selected for the subsequent experiments.

### 3.2. Optimal Reaction Temperature Selection for RT-RAA

The RT-RAA assay was performed by using the FMDV-O-F4/R4 primer set and FMDV serotype O RNA as the template. The results of agarose gel electrophoresis indicated that the amplification band was the brightest and most distinct at a reaction temperature of 37 °C (Figure 2B). Therefore, the 37 °C temperature was chosen as the optimal reaction temperature for RT-RAA.

### 3.3. crRNA Selection for RT-RAA-CRISPR/Cas13a

**Figure 3:**
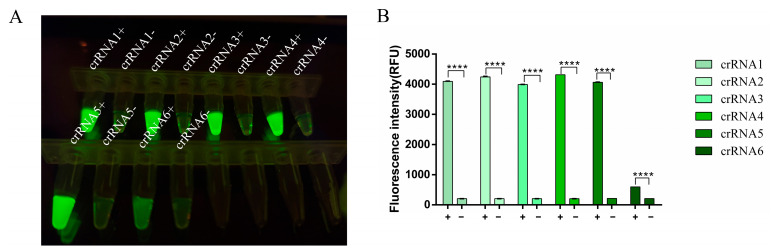
crRNA selection for the RT-RAA–CRISPR/Cas13a assay. The CRISPR/Cas13a assay was performed by using six designed crRNAs. +: FMDV serotype O RNA template was added. −: negative control (nuclease-free water) was added. (**A**) The fluorescence signal was detected by using a blue light transmission instrument. (**B**) The fluorescence intensities were measured by using a multifunction microplate reader. Each column indicates the mean of the triplicate fluorescence values ± SDs, and **** indicates a significant difference (*p* < 0.001).

Six specific crRNA sequences were designed for the FMDV-O-F4/R4 amplification sequence. The RT-RAA-CRISPR/Cas13a reactions were conducted with different crRNAs. The results indicated that crRNA4 produced the highest fluorescence intensity (Figure 3). Therefore, crRNA4 was selected as the optimal crRNA for this study.

### 3.4. Specificity Testing for RT-RAA-CRISPR/Cas13a Assay

**Figure 4:**
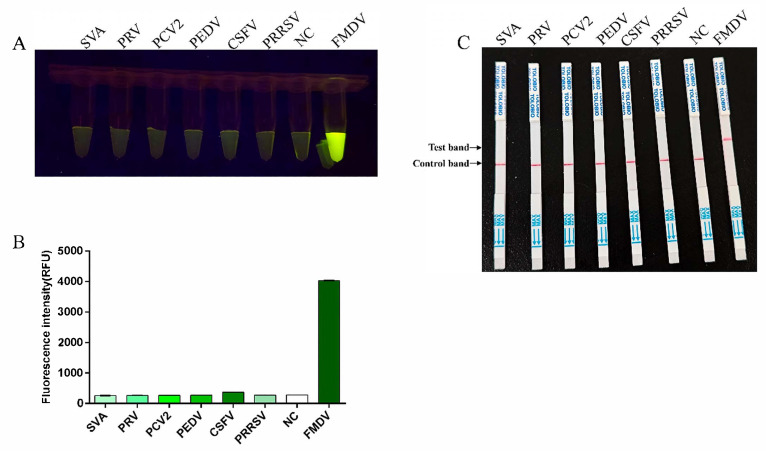
Specificity of RT-RAA-CRISPR/Cas13a. (**A**) The fluorescence signal was detected by using a blue light transmission instrument. (**B**) The fluorescence intensities were measured using a multifunction microplate reader. (**C**) Specificity examination via RT-RAA-CRISPR/Cas13a LFS.

The RT-RAA-CRISPR/Cas13a assay was performed by using nucleic acids from six common swine viruses, including CSFV, PEDV, PCV2, PRV, PRRSV, and SVA to evaluate the specificity of this method. The fluorescence was detected in the reaction tube only when the FMDV serotype O RNA was used as the template. Fluorescence was unobserved in the other reaction tubes, thereby indicating that the established RT-RAA-CRISPR/Cas13a method had excellent specificity (Figure 4A,B). Consistent with these findings, the LFS demonstrated the same results (Figure 4C).

### 3.5. Sensitivity Testing for RT-RAA-CRISPR/Cas13a Assay

**Figure 5:**
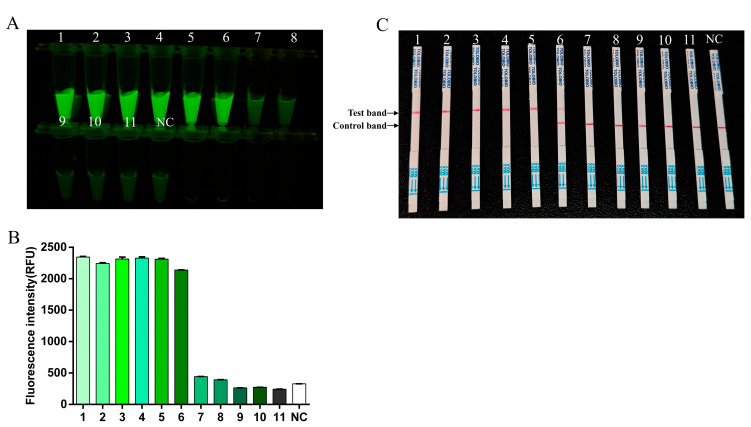
The sensitivity of RT-RAA-CRISPR/Cas13a. 1–11: template dilution ranges from 1 × 10^0^–1 × 10^−10^. NC, negative control. (**A**) The fluorescence signal was detected by a blue light transmission instrument. (**B**) The fluorescence intensities were measured using a multifunction microplate reader. Each column indicates the mean of the triplicate fluorescence values ± SDs. (**C**) Sensitivity examination via the RT-RAA-CRISPR/Cas13a LFS assay.

To determine the sensitivity of the RT-RAA-CRISPR/Cas13a method, FMDV serotype O RNA was serially diluted 10-fold and quantified by digital PCR. The copy numbers of diluted templates were 98,665 copies/µL (10^−2^), 4374.4 copies/µL (10^−3^), 221.2 copies/µL (10^−4^), and 19.1 copies/µL (10^−5^). The RT-RAA-CRISPR/Cas13a assay was performed by using the original O-type FMDV RNA standard and the first 10 dilutions of the FMDV serotype O RNA standard as templates. The fluorescence intensity and LFS were detectable up to the 10^−5^ dilution of the FMDV RNA, thus indicating a detection limit of 19.1 copies/µL for FMDV (Figure 5).

### 3.6. Repeatability Testing for RT-RAA-CRISPR/Cas13a Assay

**Figure 6:**
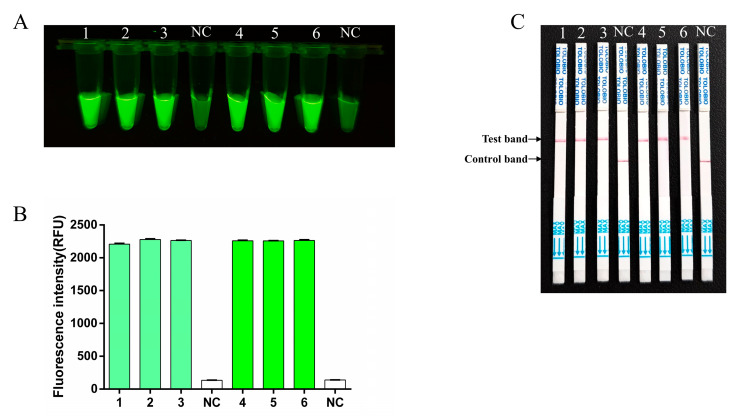
Repeatability of the RT-RAA-CRISPR/Cas13a assay. 1–3: template dilution 1 × 10^−1^; 4–6: template dilution 1 × 10^−3^, NC, negative control. (**A**) The fluorescence signal was detected by a blue light transmission instrument. (**B**) The fluorescence intensities were measured using a multifunction microplate reader. Each column indicates the mean of the triplicate fluorescence values ± SDs. (**C**) Repeatability tests via the RT-RAA-CRISPR/Cas13a LFS assay.

The repeatability of the RT-RAA-CRISPR/Cas13a method was assessed by conducting three independent assays for the 10^−1^ and 10^−3^ dilutions of the FMDV serotype O RNA template, with each assay being repeated three times. Fluorescence was consistently produced in all three independent trials (Figure 6A,B). The LFS demonstrated the same results (Figure 6C), thus indicating that the established method had the ideal repeatability.

### 3.7. Simulated Clinical Sample Detection for RT-RAA-CRISPR/Cas13a Assay

**Figure 7:**
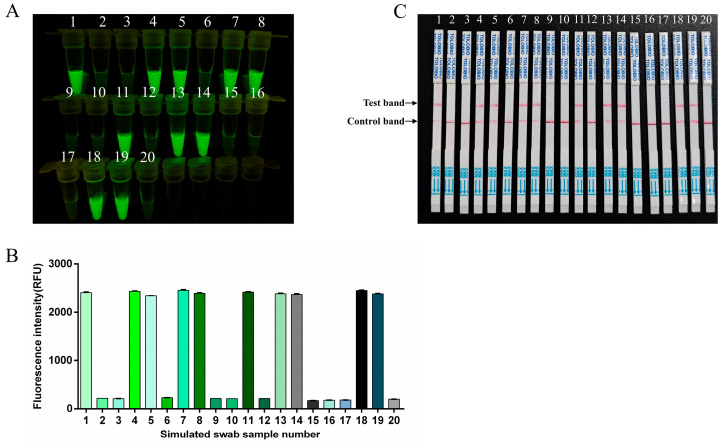
Simulated swab sample analysis. 1–20: simulated swab samples. (**A**) The fluorescence signal was detected by a blue light transmission instrument. (**B**) Fluorescence intensities were measured using a multifunction microplate reader. (**C**) RT-RAA-CRISPR/Cas13a LFS detection results for simulated swab samples.

**Figure 8:**
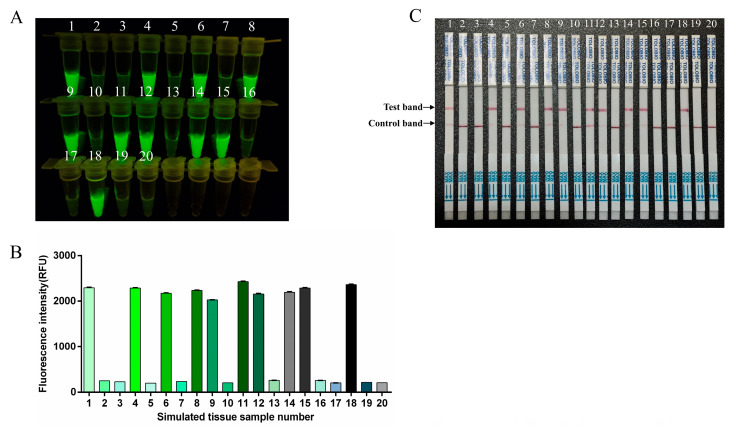
Simulated tissue sample analysis. 1–20: simulated tissue samples. (**A**) The fluorescence signal was detected by a blue light transmission instrument. (**B**) Fluorescence intensities were determined using a microplate reader. (**C**) RT-RAA-CRISPR/Cas13a LFS detection results for simulated tissue samples.

**Figure 9:**
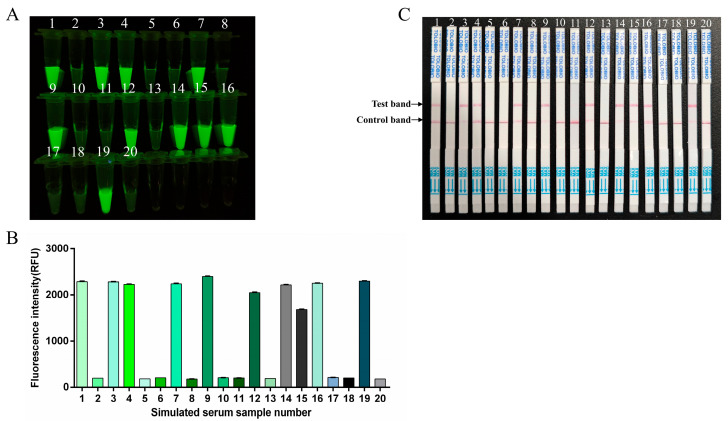
Simulated serum sample analysis. 1–20: simulated serum samples. (**A**) The fluorescence signal was detected by a blue light transmission instrument. (**B**) Fluorescence intensities were measured using a multifunction microplate reader. (**C**) RT-RAA-CRISPR/Cas13a LFS detection results for simulated serum samples.

As FMDV clinical samples were difficult to obtain, the simulated clinical samples were used to evaluate the performance of the RT-RAA-CRISPR/Cas13a method for sample detection. Ten simulated FMDV-positive clinical samples and ten simulated FMDV-negative clinical samples for each type (swab, tissue, and serum samples) of simulated clinical sample were blindly tested with this method, and the results were compared with the original preparation results. Both the fluorescence and lateral flow results indicated that ten of the twenty reactions produced positive signals, which was consistent with the sample preparation (Figure 7, Figure 8 and Figure 9).

## 4. Discussion

FMDV is currently considered to be a worldwide pathogen and poses a significant threat to the global swine industry [27]. Infected pigs can harbor the virus for extended periods. Live pig trade facilitates its transmission between farms [28,29]. Additionally, the vesicular lesions caused by FMDV can be mistaken for those caused by other pathogens, such as SVA and SVDV, thereby leading to misdiagnosis and heightened anxiety among farmers [30]. Therefore, rapid and accurate onsite diagnostic methods for FMDV are urgently required in the swine industry. Conventional laboratory diagnostics is impractical for use on small- and medium–sized pig farms because of the lack of expensive laboratory equipment and trained personnel. Large-scale farms also experience various challenges such as the need to use shared laboratories and delayed sample submission, which can hinder timely disease control [31]. In this study, we developed a new method for detecting the FMDV serotype O. This method can amplify low-copy samples under isothermal conditions with a minimal requirement for equipment, while ensuring high specificity and sensitivity. Moreover, it is simple to perform, and requires only a portable isothermal fluorescence detector, additionally, the reagents used can be lyophilized, eliminating reliance on cold chain transportation. Thus, this method is suitable for onsite diagnosis in the field.

The SHERLOCK technique, which emerged in 2017, integrates RT-RAA and CRISPR/Cas13a techniques to increase detection sensitivity and specificity [22]. The principle of this method involves the use of RT-RAA primers containing a T7 promoter to amplify the pathogen sequence, which is then transcribed to RNA to increase the amount of template available for detection [32]. The collateral cleavage activity of the Cas enzyme, which is activated by crRNA-guided recognition of specific RNA sequences, leads to cleavage of the reporter probe to generate a signal. Unlike Cas12a, which requires a protospacer adjacent motif (PAM) sequence for target recognition, Cas13a is not involved in this requirement [33]. Several pathogens have been detected using RT-RAA and CRISPR/Cas13a techniques, such as SARS-CoV-2 in wastewater by Yang et al. [32]. This method addresses the technical gap associated with the inability of digital PCR to detect viral nucleic acids in wastewater, thereby validating the practicality of this method for environmental water samples [32]. Liu et al. developed an onsite detection method for H7N9 avian influenza using this technique, whereby they achieved detection limits of 1 fM for hemagglutinin and 1 nM for single-stranded RNA within 5 min with high specificity [34]. In addition, the one-pot RPA-CRISPR/Cas13a assay for NiV detection exhibited specificity and did not demonstrate cross-reactivity with other selected emerging pathogens [26].

In this study, a poly U RNA fluorescence probe was designed on the basis of the cleavage preference of the LwaCas13a protein. The optimal crRNA was selected on the basis of the target sequence to further ensure the specificity of the method, and avoid false-positive results. The sensitivity of the method was verified, with a detection limit of 19.1 copies/µL being observed; moreover, no cross-reactions with other common swine pathogens, such as SVA, PEDV, CSFV, PCV2, PRV, and PRRSV, were observed. However, due to the extremely high sensitivity of the RT-RAA-CRISPR/Cas13a method, numerous factors can influence fluorescence, thus making it difficult to assess repeatability by conventional means using CV values. Repeatability can only be evaluated on the basis of the consistent detection of fluorescence and the LFS results [35]. Additionally, CRISPR detection methods can be adapted into lateral flow test strip-based methods for onsite use, whereby the cleaved reporter probe can be captured on a detection line, thus producing a visually observable band [36]. The results of simulated clinical sample testing revealed that the established method is efficient for detecting porcine samples in situ.

However, the established method still has several limitations. First, given the significant sequence variations observed among FMDV strains across different global regions and considering that the primary objective of this study was to develop a point-of-care diagnostic test for porcine O-type FMDV in China, all of the O-type strain sequences from China detected during the past 14 years (2010–2024) (including subtypes such as CATHAY, SEA, India2001, and PanAsia, along with several representative sequences of epidemic strains from neighboring countries) were collected for analysis. The conserved sequences identified via comparative analysis of these long-term evolved subtypes with substantial mutational differences are presumed to represent relatively stable regions of the FMDV genome. Although this strategy maximizes the detection capability for currently prevalent strains in Chinese swine populations, it may fail to identify strains that have been introduced from regions outside of Southeast Asia, thereby potentially leading to missed detections and false-negative results. Second, due to the difficulty in obtaining FMDV-positive clinical samples, this study utilized simulated clinical samples (such as swabs) for method validation. This approach may introduce discrepancies between the test results and those results obtained from authentic samples, thereby necessitating further validation with clinical samples in future studies. Finally, the current detection process requires the uncapping of the reaction tubes for lateral flow testing, during which amplified product exposure may generate aerosol contamination in the testing environment, thus potentially causing false-positive results in subsequent experiments. To address this issue, we are currently developing an integrated detection device that physically isolates nucleic acid amplification and detection units, thereby enabling closed “sample-in, result-out” detection. This modification is expected to effectively mitigate contamination risks. We will continuously refine these limitations to increase the applicability and reliability of this method, thereby ultimately providing a more advanced and user-friendly diagnostic solution for porcine O-type foot-and-mouth disease.

## 5. Conclusions

A rapid RT-RAA-CRISPR/Cas13a detection method for detecting FMDV serotype O in swine has been successfully established in this study. This method is highly sensitive, with a detection limit of 19.1 copies/µL, and exhibits excellent specificity, with no cross-reactions with other common swine pathogens being observed. Moreover, the assay can be completed within 1 h, thus providing a new onsite diagnostic method for swine FMDV serotype O.

## Figures and Tables

**Table 1:** RT-RAA primers, crRNA, and probes.

Primer Name	Primer Sequence (5′ → 3′)
FMDV-O-F1	GAAATTAATACGACTCACTATAGGGGACCCAMCATGTGTGCAACCCC
FMDV-O-R1	CCGGTCRCCTATTCAGGCATAGAAGC
FMDV-O-F2	GAAATTAATACGACTCACTATAGGGCTATGAGAACAAACGCATYACMGTTGARG
FMDV-O-R2	GTAGATGTTGTTCARAATTGTGTTGATGAT
FMDV-O-F3	GAAATTAATACGACTCACTATAGGGCTTYAAATCYCTTGGYCAAACCATYACTCC
FMDV-O-R3	TTTGTAAAACCCAGTTCCATARTCCATGTG
FMDV-O-F4	GAAATTAATACGACTCACTATAGGGAACAACAARTTGGACATMAYCAAAGCTC
FMDV-O-R4	GAACAYRTCTTGTTGCATTCTCTTCATTTC
FMDV-O-F5	GAAATTAATACGACTCACTATAGGGTCGAAGACCCTCGARGCYATCCTCTC
FMDV-O-R5	CGTTCACCCADCGCAGGTAAAGTGA
FMDV-O-F6	GAAATTAATACGACTCACTATAGGGCAAACCATYACTCCAGCTGACAAAAGC
FMDV-O-R6	ATCACAGGTTTRTAAAACCCAGTTCCRTA
crRNA Template 1	CARTAYGACTGTGCCCTTCTCAACGGCAGTTTTAGTCCCCTTCGTTTTTGGGGTAGTCTAAATCCCCTATAGTGAGTCGTATTAATTTC
crRNA Template 2	AATAYGACTGTGCCCTTCTCAACGGMATGTTTTAGTCCCCTTCGTTTTTGGGGTAGTCTAAATCCCCTATAGTGAGTCGTATTAATTTC
crRNA Template 3	ATAYGACTGTGCCCTTCTCAACGGMATGGTTTTAGTCCCCTTCGTTTTTGGGGTAGTCTAAATCCCCTATAGTGAGTCGTATTAATTTC
crRNA Template 4	TAYGACTGTGCCCTTCTCAACGGMATGGGTTTTAGTCCCCTTCGTTTTTGGGGTAGTCTAAATCCCCTATAGTGAGTCGTATTAATTTC
crRNA Template 5	AYGACTGTGCCCTTCTCAACGGMATGGCGTTTTAGTCCCCTTCGTTTTTGGGGTAGTCTAAATCCCCTATAGTGAGTCGTATTAATTTC
crRNA Template 6	YGACTGTGCCCTTCTCAACGGMATGGCCGTTTTAGTCCCCTTCGTTTTTGGGGTAGTCTAAATCCCCTATAGTGAGTCGTATTAATTTC
T7-oligo	GAAATTAATACGACTCACTATAGGG
Fluorescence RNA reporter	5′-FAM/UUUUUUUUUUU/BHQ1-3′
LFS RNA reporter	5′-FAM/UUUUUUUUUUU/Biotin-3′
FMDV 3D F	GCGAGTCCTGCCACGGA
FMDV 3D R	ACTGGGTTTTACAAACCTGTGA
FMDV 3D probe	5′-FAM/TCCTTTGCACGCCGTGGGAC/BHQ1-3′

Note: The underlined sequence represents the T7 promoter.

## Data Availability

The data presented in this study are available upon request from the corresponding author.

## Supplementary Materials

The following supporting information can be downloaded at: https://www.mdpi.com/article/10.3390/v17050721/s1, Figure S1: NCBI reference sequence of the FMDV complete genome; Table S1: Sequence alignment results.
