# Supplementary material for: Establishment and Implementation of the Point-of-Care RT-RAA-CRISPR/Cas13a Diagnostic Test for Foot-And-Mouth Disease Virus Serotype O in Pigs
Source: Viruses. 2025 May 17;17(5):721. doi: 10.3390/v17050721 (PMC12115470; doi:10.3390/v17050721)
Supplement: Supplementary file 1 [file viruses-17-00721-s001.zip › viruses-3586629-supplementary.pdf]

Table S1. NCBI reference sequence of the FMDV complete genome

Definition	NCBI Reference Sequence	Serotype
Foot-and-mouth disease virus O isolate GD/CHA/JH12/2013 complete genome	KU204894.1	O
Foot-and-mouth disease virus O, complete genome	MN389541.1	O
Foot-and-mouth disease virus O isolate O/XJ/CHA/2017, complete genome	MF461724.1	O
Foot-and-mouth disease virus O strain O/GD/CHA/2015, complete genome	KY234502.1	O
Foot-and-mouth disease virus - type O polyprotein gene, complete cds	AH012984.2	O
Foot-and-mouth disease virus - type O strain O/BY/CHA/2010, complete genome	JN998085.1	O
Foot-and-mouth disease virus O isolate HN/CHA/HK212/2013 polyprotein gene, complete cds	KU204893.1	O
Foot-and-mouth disease virus O strain O/GC/SKR/2016, complete genome	KY086466.1	O
Foot-and-mouth disease virus O isolate G12_1_SJSP_0416/Ratchaburi/Thailand/2016, complete genome	KY444647.1	O
Foot-and-mouth disease virus O isolate O/VIT/20/2011 polyprotein gene, partial cds	MF947123.1	O

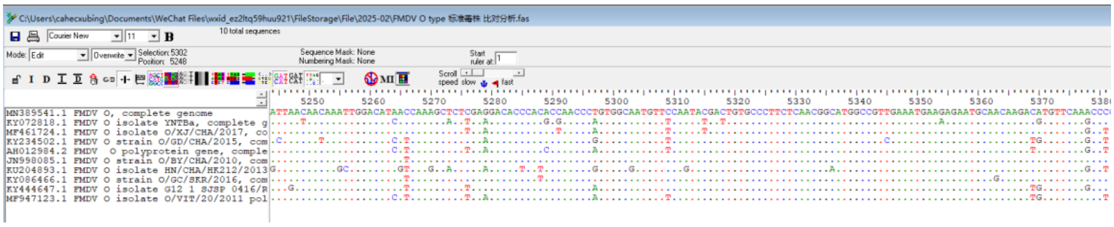

Figure S1. Sequence alignment results
